# 

**Published:** 1867-09

**Authors:** 


### PERSONAL.

Our readers will be pleased to know that our friend Mr. James Leslie, is again at his post after a pleasant visit to Europe. He was, of course, at the World's Fair, also in England, Scotland, etc. He could tell us much about the state of the profession in Europe, and when he gets his private matters straightened up a little we hope he will do so in the pages of the Register. In the meantime you can call and hear him speak for himself.

### CONTENTS.

### The Dental Register

### OF THE WEST.

*Issued Monthly, at $3 a Year in Advance.*

DEVOTED TO THE INTERESTS OF THE DENTAL PROFESSION.

EDITED BY J. TAFT AND G. WATT.

The volume commences in January and closes in December.

The Register being issued monthly is always fresh, therefore indispensable to the practitioner who desires to keep posted up with the new inventions and improvements which are daily developed. Neither expense nor effort will be spared to give value and variety to its contents. Articles will be illustrated with well executed engravings whenever they will add to the interest or assist in explanation.

Its extensive circulation and frequency of issue makes it one of the BEST DENTAL ADVERTISING MEDIUMS in the United States.

RATES OF ADVERTISING.

One page, 1 year, $50. Half page, 1 year, $30. Quarter page, or card, 1 year $20.

All advertising bills payable in advance, or at furthest after the issue of the first number containing the advertisement. All transient advertisements to be paid for in advance.

☞ CONTRIBUTIONS SOLICITED.

Address, J. TAFT, or "DENTAL REGISTER," Cincinnati, Ohio.

Business Communications to

**J. TAFT, Publisher,**

No. 238 Fourth St., between Plum and Central Avenue

